# The Diagnosis and Treatment of Typhoid Fever

**Published:** 1896-05

**Authors:** B. F. Brittain

**Affiliations:** Arlington, Texas


For the Texas Medical Journal.

### THE DIAGNOSIS AND TREATMENT OF TYPHOID FEVER.

B. F. BRITTAIN, M. D., ARLINGTON, TEXAS.

[Read before the East Texas Medical Association, April 14, 1896.]

THERE is not much liability to confound typhoid fever with any other disease in Texas but malarial fever. I have long entertained the notion that there were only two distinct continued fevers in Texas, viz: malarial fever, when it assumes the continued form, and the typhoid fever. The so-called slow fever is a misnomer. It is the name of no disease at all. We gave some of the continued fevers that name when we were in doubt as to what to call a fever that was prevailing, not only in Texas, but all over the South. It was wanting in many of the distinctive symptoms of typhoid fever, but some calling it typhoid, some malarial fever, some contending it was neither malarial fever nor typhoid, but that it was entirely different. During this period of uncertainty Dr. C. H. Wilkison, of Galveston, wrote Prof. Flint, of New York, detailing the symptoms of the supposed new fever, and his answer was that it was typhoid fever without doubt. This to a great extent settled the dispute as to the nature of this atypical form of typhoid fever. Prof. West, of Galveston, about the same time, was conducting an investigation on this line and found in autopsies, if I mistake not, the characteristic lesions of Peyer's glands.

In making my diagnosis of typhoid fever, I have, in a measure, quit depending on the symptoms and diagnostic points as detailed in the text-books, for they are often misleading, and you are just about as liable to make a wrong as a right diagnosis. The best general plan is, to go slow,—tell your patient, who will perhaps importune you for a definite statement as to the nature of his fever, that the type is not fully set—that it usually takes a few days—that, as a rule, for the first few days the fever produces only a general pathological condition, and, during that time, it is only needed to resort to general therapeutics, which is my main reliance in making my diagnosis.

Osler acknowledges in his practice that he has more than once made a wrong diagnosis between the malarial and typhoid fevers. He has sent patients with typhoid fever to the malarial wards, and vice versa, he has sent patients with malarial fever to the typhoid wards, and afterward found out his mistakes and frankly admitted it. Honor to his name for his honesty.

The surest way to make the diagnosis is to examine the blood under the microscope before you give any quinine, for quinine disorganizes the malarial plasmodium. But the microscope is not a practicable means for the simple reason that few of us own a microscope, and still fewer are experts in its use.

The best way to make the diagnosis is to give calomel and quinine for four or five days, and, if it is malarial fever, you will either cure your patient or greatly mitigate his symptoms, and, if it is typhoid, you will not be able to make any impression on the disease, but the fever will be apt to be a little higher from day to day for a week or more!

Now, gentlemen, I have given you my mode of finding out, whether a given case is typhoid fever or something else.

*Treatment.*—Without consuming time to mention all the various treatments heretofore in vogue for typhoid fever, permit me to say the present is universally acknowledged, viz: the antiseptic treatment, and as it is a self-limited disease, it is best to select non-spoliative, non-perturbating, remedies, especially after four or five days. While making your diagnosis, use calomel and quinine for about five days, then stop their use, and as a rule, use them no more during the course of the disease.

I have quit using the German method—of using iodine as modified by Bartholow, which is two drops of the tr. iodine and one of carbolic acid every few hours. To most patients it is a disgusting mixture and has no control of the disease. Sulpho-carbolate of zinc, in two to five grain doses, every two hours, is highly spoken of by Waugh. I have used it some, but not enough to pass jndgment on it; salol is used by many practitioners. I have used it but can't say that I am especially delighted with it. I frequently use the old-time remedy, turpentine. I use water as a therapeutic remedy, not like Brand uses it—which, you know, is the cold bath—nor do I use the luke-warm bath and gradually cooled by adding cold water, because it is inconvenient. I sponge the body, as recommended by Osler, either with cold or tepid water as suits the patient's feelings best. The sponging should be done every hour when the temperature is as high as 102½° F. If the temperature should reach 103° I direct a systematic sponging, which is performed by taking a limb at a time and thoroughly sponge it, and then the breast, abdomen, head and neck; then, turning on the side, sponge the back thoroughly. It takes about twenty minutes to go through this sponging properly. It invariably reduces the fever and quiets the nerves.

For internal medication I prefer the chlorine mixture to any other antiseptic remedies, and you can make it yourself as follows, viz:

℞ Chlorate potash, pulv., gr. 20.
Hydrochloric acid, gtt 60.

Mix in a pint bottle, and in five to ten minutes the chlorine gas will be evolved, when you can add, gradually, 15 oz. water, shaking bottle every three or four ounces added. Now add about

1 oz. syr. orange, and you have a nice, palatable mixture, which you can give to your patient in two to four tablespoonfuls from one to two or three hours apart, accordingly, as the discharges from bowels are offensive or otherwise.

